# Femoral head penetration in Vitamin-E polyethylene liner versus conventional liners in total hip arthroplasty: systematic review and meta-analysis of randomised control trials

**DOI:** 10.1051/sicotj/2021045

**Published:** 2021-09-10

**Authors:** Hany Elbardesy, Himanshu Yadav, Mohamed Rabea, Shane Guerin, James Harty

**Affiliations:** 1 Department of Trauma and Orthopaedic, Cork University Hospital Wilton Cork T12DFK4 Ireland; 2 Faculty of Pharmacy, Mansoura University Mansoura 32216 Egypt

**Keywords:** Primary hip replacement, Vitamin-E, Polyethylene, Radio stereometry, Head penetration, Meta-analysis

## Abstract

*Background*: Debate encompasses the use of Vitamin E Polyethylene or conventional Polyethylene liner in primary hip arthroplasty. Does the Inclusion of Vitamin E in PE give adequate protection from oxidation and maintains lower rates of wear? *Patients and methods*: We performed this study following the Preferred Reporting Items for Systematic Reviews and Meta-analyses Statement (PRISMA) and the Cochrane Handbook for systematic reviews and meta-analysis. Studies were included from any region, written in any language. We had only the randomised control trials comparing the femoral head penetration between Vitamin-E diffused highly cross-linked polyethylene (VEPE) liner and conventional liners in primary total hip arthroplasty. *Results*: We included 10 studies in this meta-analysis. We conducted them using Review Manager V.5.0. We computed the risk ratio to measure the treatment effect, considering the heterogeneity. We used Random-effect models. VEPE had insignificant marginal advantages for FHP within three months post-operative. Additionally, VEPE showed significantly less FHP after two and five years. After one year, it showed significantly less FHP with the VEPE group versus the UHMWPE cohort and a non-significant difference between the VEPE and XLPE group. *Conclusions*: In terms of FHP, this metanalysis shows less FHP for the VEPE than conventional PE. A longer follow-up period is required to evaluate whether the oxidation protection gained by Vitamin E results in lower wear rates, less osteolysis, and aseptic loosening compared to the conventional PE in the long term.

## Introduction

For the last decades, the development of polyethylene (PE) manufacturing has led to a remarkable reduction in the number of revision hip arthroplasty caused by periprosthetic osteolysis due to the debris particulate of PE [1–3]. The ultrahigh-molecular-weight polyethylene (UHMWPE) liner in total hip arthroplasty (THA) started in 1962. Although it achieved remarkable clinical success, PE wear was a major concern. Thus, the development of highly cross-linked polyethylene (HXLPE) happened in the later 1990. Nevertheless, the concerns related to mechanical properties and oxidation of the HXLPE lead the development of second-generation (sequentially annealed, mechanically annealed, and Vitamin-E containing) HXLPE [4]. Adding Vitamin E to PE gives extra protection from oxidation and maintains low wear rates. Additionally, it also has a beneficial effect to reduce the inflammatory reaction to the particles associated with wear [5]. Various microorganisms demonstrated a reduction of the adherence properties to the Vitamin E-incorporated ultra high molecular weight polyethylene (UHMWPE) [4]. Crosslinking is accomplished by irradiating PE at a higher dose than the required amount for sterilization [5]. This process leads to the development of covalent bonds between the polymer chains of PE, which gives extra protection from wear [6]. The resulting polymer is rich in free radicals. Therefore, it is sensitive to oxidation, making it more brittle and less wear resistant. The blending process starts by dissolving the Vitamin E in isopropanol in a 20 g/L concentration in one kg batches. The UHMWPE powder then manually mixed with the Vitamin E/isopropanol solution. The blend was dried afterward in a convection oven under vacuum at 60 °C for one week [4]. The blend is then irradiated for cross-linking, machined into components, and packaged [9]. The advantage of diffusion over blending is related to the fact that according to the amount of Vitamin E blending, cross-linking can be reduced due to the presence of Vitamin E. The advantage of blending is that Vitamin E is homogenously distributed into polyethylene. In contrast, diffusion provides mainly Vitamin E at the articulating surface and not into the core of the material [5].

Annealing could increase the oxidative stability of PE by reducing the free radical content by heating the PE below its melting point [7]. Annealing could also be achieved mechanically by hydrostatic extrusion. It maintains the mechanical strength, however, it traps the free radicals within the crystal domains of the PE [8]. Vitamin E effectively reduces the remaining free radicals after the irradiation process, without the need for heating after irradiation [9, 10]. Therefore, Vitamin E embedded PE enhances the long-term resistance to oxidation by maintaining its mechanical properties [11–13]. Currently, a variation of Vitamin E Polyethylene (VEPE) is widely available on the market nation widely [11], and the current results of VEPE in primary THA are promising [14, 15]. This meta-analysis aims to evaluate studies assessing femoral head migration after three months, two, and five years for both VEPE and conventional PE [X linked polyethylene (XLPE) or ultra high molecular weight polyethylene (UHMWPE)].

## Materials and methods

**Figure 1:**
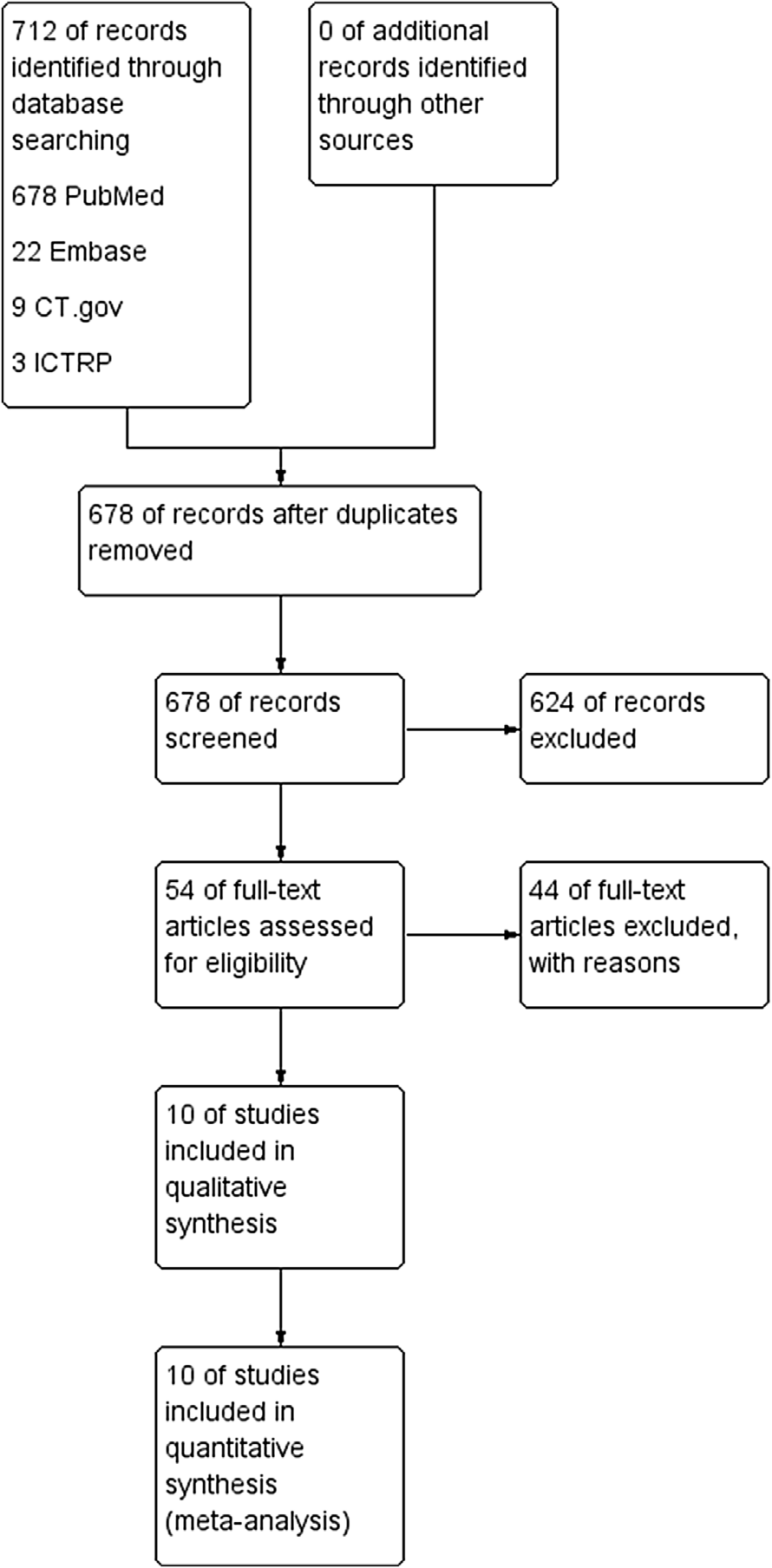
Preferred reporting items for systematic reviews and meta-analyses (PRISMA) flow chart.

We performed this study following both the Preferred Reporting Items for Systematic Reviews and Meta-analyses Statement (PRISMA) (Figure 1) and the Cochrane Handbook for systematic reviews and meta-analyses [16]. We conducted an initial search using the MEDLINE-OVID, Web of Science, PubMed, EMBASE-OVID, Google Scholar, and Cochrane Library. Grey and unpublished literature were also explored by searching: Grey Matters BIOSIS Previews, International Clinical Trial Registry, UK Clinical Trials Gateway, https://ClinicalTrials.gov, Networked Digital Library of Theses and Dissertations, UK Clinical Research Network Study Portfolio, Open Grey and Grey Literature Report. We used the following keywords and their combinations: Total hip arthroplasty, Vitamin-E, Polyethylene, Radio Stereometric Analysis (RSA), and head penetration. Articles published up to December 2020 were included in our literature search and were limited to studies in human subjects published in any language. Additionally, we cross-referenced the bibliographies of retrieved articles and review papers to ensure that we captured all relevant studies.

## Eligibility criteria

The inclusion criteria included

1. Two arms randomised control trials (RCTs) compared the VEPE liners and conventional UHMWPE or XLPE liners.
2. Total head migration reported by using RSA.
3. At least two years of follow up.

We excluded all studies that did not meet one or more of the eligibility criteria.

## Methodological quality assessment

**Figure 2:**
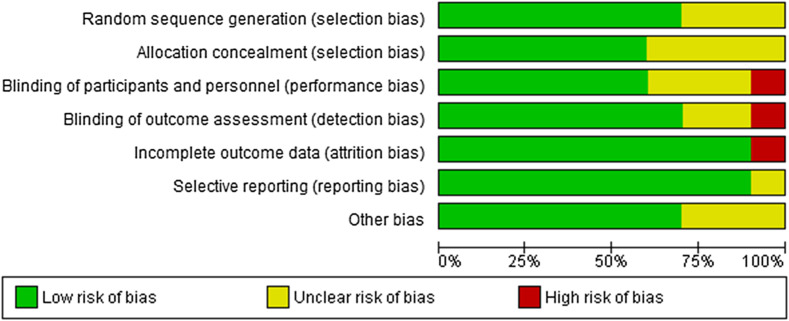
Risk of bias graph: review authors’ judgements about each risk of bias item presented as percentages across all included studies.

**Figure 3:**
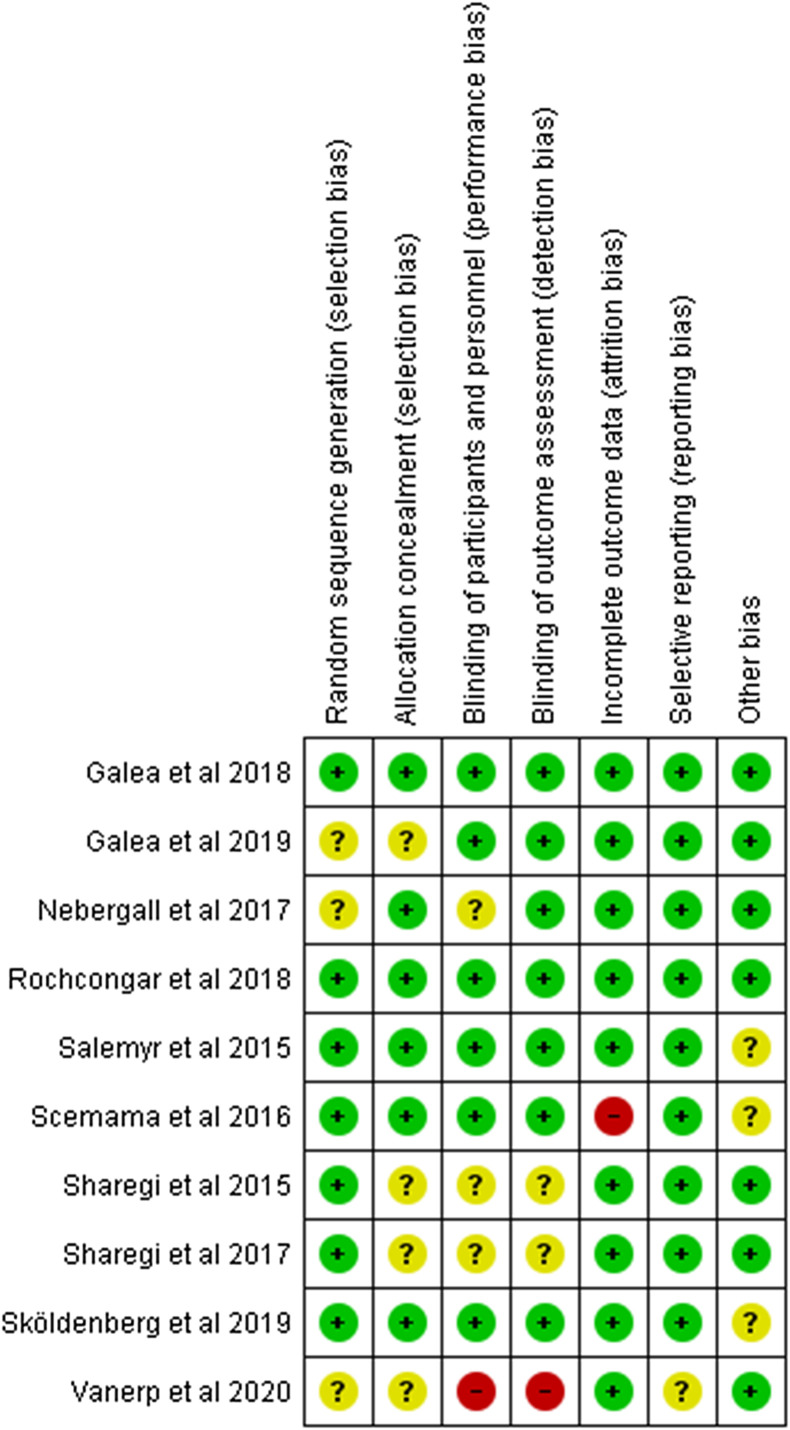
Risk of bias summary: review authors’ judgements about each risk of bias item for each included study.

We assessed the risk of bias (Figures 2 and 3) for included studies by using the Cochrane risk of bias criteria [16] and independently cross-checked by the five reviewers (HE, HY, MR, SG, and JH). Disagreements were resolved through discussion.

## Risk of Bias (ROB) assessment

The methodological quality varied between the RCTs (Figure 2). Randomization was adequate in five trials [15, 17–20], with two trials estimated to have some concerns or be at high ROB [21, 22]. Outcome data were adequate in nine studies, with one study reporting higher rates of attrition bias as a result of losses to follow-up [20]. Overall bias assessment was considered sufficient in eight of the 10 RCTs [15, 17–19, 21–24].

## Data extraction and synthesis

Two authors (HE and MR) independently screened all titles and abstracts identified by the initial search to assess their eligibility for inclusion. Then we did a full screening of the manuscript and conducted a final assessment of the eligibility for all included studies. Then the same two reviewers did data extraction. Any discrepancies after data collection were resolved by discussion between all reviewers. The collected information included first author, year, journal, Country, level of evidence, type of study, number of centres, study length, numbers, age, gender, and Body Mass Index (BMI).

## Outcome measures

The primary outcome of interest was Femoral Head Penetration (FHP) after two years postoperative, the second outcome measure was FHP after three months, one and five years.

### Statistical analysis

We conducted a statistical analysis by using Review Manager (RevMan), version 5.3 (The Nordic Cochrane Centre, The Cochrane Collaboration, 2009, Copenhagen, Denmark) [25]. Heterogeneity between studies was assessed by the *I*^2^ statistic, and a *c*^2^ of <0.05 was used to define the significance of the heterogeneity among the included studies, ranges of 0–24%, 25–74%, and 75–100% were considered minor, moderate and major heterogeneity respectively [16]. Mean differences and standard deviations (SDs) were used for continuous variables. We used the Mantel-Haenszel random-effects model in our meta-analysis. We illustrated the results using forest plots, a 95% confidence interval (CI) for each study, and a cumulative weighted mean difference (MD) for all the included studies. Besides this [16].

## Results

### Study characteristics

Our literature search revealed 712 unique references. After reviewing all the studies’ titles and abstracts, 10 studies were eligible for both quantitative and qualitative analysis. A summary of the patient’s demographics is presented in Table 1.

**Table 1:** Studies characteristics.

Study	Country	Journal	Study type	Centers	Level of evidence	Type of PE
Sköldenberg et al. [17]	Sweden	BJJ	RCT	1	1	UHMWPE
Galea et al. [23]	International	BJJ	RCT	4	1	XLPE
Galea et al. [24]	USA	BJJ	RCT	1	1	XLPE
Nebergall et al. [15]	Denmark	BJJ	RCT	1	1	XLPE
Rochcongar et al. [18]	France	JBJ S	RCT	1	1	UHMWPE
Salemyr et al. [19]	Sweden	International Orthopaedics (SICOT)	RCT	1	1	XLPE
Scemama et al. [20]	France	International Orthopaedics (SICOT)	RCT	1	1	UHMWPE
Shareghi et al. [21]	Sweden	JBJ S	RCT	1	1	XLPE
Shareghi et al. [22]	Sweden	JBJS	RCT	1	1	XLPE
van Erp et al. [31]	Netherlands	Acta orthopaedica	RCT	1	1	UHMWPE

Studies characteristics, RCT: Randomised Controlled Trial; BJJ: Bone and Joint Journal; JBJS: The Journal of Bone and Joint Surgery.

### Patient baseline characteristics

The subjects in the VEPE cohort had an average age of 63.7 years (± 10.25 years), of which 277 out of 519 patients (53.37%) were female, with an average body mass index of 26.77 kg/m^2^ (± 5.85). The conventional PE cohort had a similar patient distribution with an average age of 63.02 years (range: ± 9.36 years), of which 227/424 (53.53%) were male, with an average body mass index of 27.06 kg/m^2^ (± 6.58), (Tables 1 and 2).

**Table 2:** Patient’s demographics.

Study	Number of patients		BMI		Gender (Female/Male)		Age (SD)	
	VEPE	PE	VEPE	PE	VEPE	PE	VEPE	PE
Sköldenberg et al. [17]	21	21	27 (4)	27 (4)	11/10	10/11	67 (4),	67 (5)
Galea et al. [23]	136	57	27.7 (4.5)	27.5 (4.2)	55/81	26 /31	59.8 (10.3)	60.8 (8.2)
Galea et al. [24]	44	45	27.2 (3.7)	28.3 (4.3)	17/22	14/20	66.1 (6.5)	62.6 (8.3)
Nebergall et al. [15]	32	35	27 (20 – 35)	27 (22 to 45)	16/16	16/19	67 (43 - 76)	65 (40-73)
Rochcongar et al. [18]	33	29	27.3 ± 4.1	26.7 ± 3.7	16/17	17/12	60.6 (6.5)	60.8(7.8)
Salemyr et al. [19]	25	26	28 ± 4	27±4	14/11	15/11	62 (6)	62(5)
Scemama et al. [20]	50	50	25 (18–37)	26 (17–32)	56/44	48/52	67 (32–74)	66 (49–75)
Shareghi et al. [21]	38	32	25 (19–38)	27 (19 to 36)	16/22	17/15	58 (32 to 75)	58 (36 to 67)
Shareghi et al. [22]	38	32	NA	NA	NA	NA	NA	NA
van Erp et al. [31]	102	97	NA	NA	77/25	64/33	66 (5)	65 (5)

SD: standard deviation; NA: Not applicable; BMI: Body Mass Index.

## Meta-analysis

Our meta-analysis comparatively assessed the FHP between VEPE and conventional PE in THA after three months, two, and five years.

### Femoral head penetration in two years

**Figure 4:**
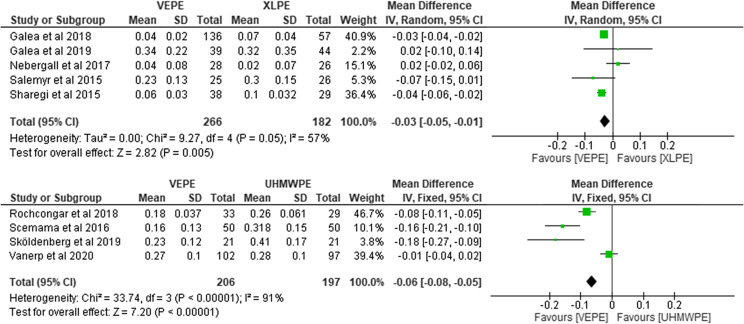
Forest plot comparing femoral head penetration between VEPE and conventional PE after two years.

Overall, nine studies indicating 851 liners reported on postoperative FHP after two years. Out of the 851 liners, 472 reported on VEPE and 379 on conventional PE (182 on XLPE and 197 on UHMWPE). They reported significantly less FHP with the VEPE group. For the VEPE versus XLPE comparison, the heterogeneity analysis demonstrated moderate statistical evidence for variation within the study (*I*^2^ = 57%). The cumulative MD was significant 0.03 (95% CI, 0.01–0.05; *P* = 0.05). For the VEPE versus UHMWPE comparison, the heterogeneity analysis demonstrated substantial statistical evidence for variation within the study (*I*^2^ = 91%). The cumulative MD was significant 0.06 (95% CI, 0.05–0.08; *P* < 0.001; Figure 4).

### Femoral head penetration in one year

**Figure 5:**
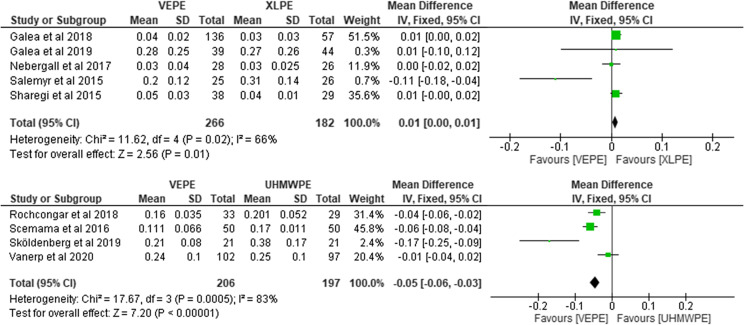
Forest plot comparing femoral head penetration between VEPE and conventional PE after one year.

Nine studies encompassing 851 liners reported on postoperative FHP after one year. Out of that number, 472 reported on VEPE and 379 on conventional PE (182 on XLPE and 197 on UHMWPE). They reported significantly less FHP with the VEPE group versus the UHMWPE cohort and a non-significant difference between the VEPE and XLPE group. For the VEPE versus XLPE comparison, the heterogeneity analysis demonstrated moderate statistical evidence for variation within the study (*I*^2^ = 66%). The cumulative MD was significant 0.01 (95% CI, 0.00–0.01; *P* = 0.02). For the VEPE versus UHMWPE comparison, the heterogeneity analysis demonstrated high statistical evidence for variation within the study (*I*^2^ = 83%). The cumulative MD was significant 0.05 (95% CI, 0.03–0.06; *P* < 0.001; Figure 5).

### Femoral head penetration in three months

**Figure 6:**
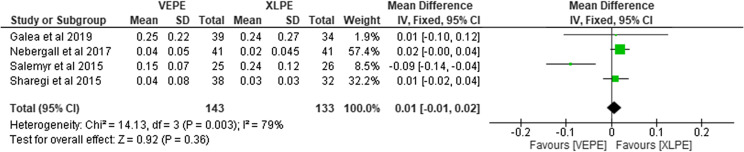
Forest plot comparing femoral head penetration between VEPE and XLPE after three months.

Four studies reported on FHP after three months post THA encompassing a total of 276 hips. Out of those, 143 were in the VEPE cohort and 133 were in the XLPE group. Two studies [15, 21] showed less FHP with the XLPE group. Heterogeneity analysis demonstrated high statistical evidence for variation within the study (*I*^2^ = 79%). Data pooled by random-effects model suggested marginally less FHP in the XLPE cohort (RR, 0.01; 95% CI, 0.02–0.04; *P* = 0.003; Figure 6).

### Femoral head penetration in five years

**Figure 7:**
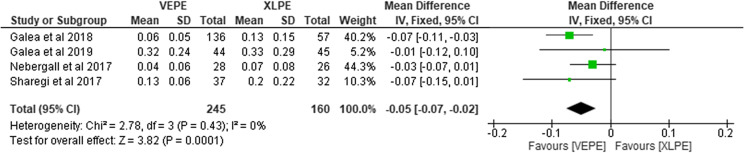
Forest plot comparing femoral head penetration between VEPE and XLPE after five years.

Four studies reported the FHP after primary THA encompassing a total of 405 liners. Among them, 245 were in the VEPE cohort and 160 in the conventional PE group. They reported significantly less FHP with the VEPE group. Heterogeneity analysis demonstrated no statistical evidence for variation within the studies (*I*^2^ = 0%). The cumulative MD was significant 0.05 (95% CI, 0.02–0.07; *P* < 0.001; Figure 7).

## Discussion

Adding an antioxidant material such as vitamin E to the irradiated PE was established to avoid reducing fatigue strength stemming from the use of other techniques such as annealing or melting. Moreover, it increases the resistance to oxidation [23]. Only one meta-analysis compared the FHP between the VEPE and XLPE but not the UHMWPE [26]. They included only five studies in their study, however, 10 studies were included in our study. Additionally, they included only two and three studies in each comparison of their meta-analysis. The manufacturing process varies between conventional PE and VEPE, and these variations include the radiation dose and post-radiation treatment. These manufacturing differences yield a balance between wear, mechanical, and resistance to oxidation, affecting their effectiveness [27]. VEPE showed lower total wear at the five-year postoperatively and a lower wear rate between two and five [23]. RSA accurately measures the FHP into the PE cup [28, 29]. Rochcongar et al. [18] argued that at three months, FHP (creep) was insignificantly less in the VEPE group than in the UHMWPE. Though, this difference was more significant at one year and became very significant at years 2 and 3. They estimated the annual wear rate for the VEPE cup by 0.020 mm/year, which is five times below the crucial value stated as a predisposing factor for osteolysis [30]. Shareghi et al. [21], at two years, found that the median proximal FHP measured 0.10 mm in the conventional PE group versus 0.06 mm in the VEPE. Salemyr et al. [19] compared the FHP between the VEPE (E1; Zimmer-Biomet) and the XLPE (Marathon™; DePuy Johnson & Jonhson, Warsaw, IN, USA). FHP was evaluated using RSA early postoperatively, and after two years, it was lower in the VEPE group. van Erp et al. [31] did not mention any complications or unusual mechanical behaviour associated with the VEPE after two years post-surgery. Some authors mentioned that the VEPE showed lower wear rates and better protection against material embrittlement and oxidation [4, 15, 20, 32, 33]. Therefore, the clinical application of VEPE could be considered safe. Many authors reported a considerable amount of FHP during the first year postoperatively. Moreover, it maintains the mechanical properties, reduces wear debris and the probability of bacterial adhesion compared to conventional PE liners. Consequently, it reduces periprosthetic loosening, and ultimately the rate of hip revision [4, 18, 20, 34]. Halma et al. [32] calculated a comparable mean FHP rate of a VEPE liner of 0.055 mm/year in 107 cups after two years. Rochcongar et al. [18] noticed a statistically significant difference (*P* < 0.001) in the FHP rate in a VEPE liner (0.020 mm/year) and a UHMWPE liner (0.058 mm/year) after three years of follow-up. Multivariate analysis showed no statistically significant difference in the wear rate with different head sizes in both cups [35, 36]. Both polyethylene types showed satisfactory midterm outcomes (5–7 years) without any significant difference in terms of Patient-Reported Outcome Measures (PROMs), for example, Harris Hip Score (HHS) [15, 22–24], SF-36 Physical Function [15, 23] and EQ-5D weighted index. Clinical evidence suggests that cup inclination angles and cup sizes correlate with PE wear, with inclination angles of ≥ 45° and cup sizes of ≥ 58 mm leading to increased PE wear with time [37, 38]. Gallo et al. [39] reported the post-traumatic THA, the underling inflammatory arthritis and the patient height as a significant factor affecting the wear rate. Moreover, they argued that the high wear rate was strongly associated with severe osteolysis. Wear has previously been suspected as a risk for late dislocation. Wear is often measured by penetration of the femoral head into the liner [38, 39]. However, the FHP is not only due to the actual loss of material (“wear”) but also due to deformation (“creep”) of the PE liner [40]. Creep occurred early after implantation and accounted for a mean penetration of 0.26 mm. It was similar for both HXLPE and UHMWPE. Almost 80% of creep occurred in the first three months and 95% by six months [40]. In conclusion, this meta-analysis shows better results of the VEPE compared with the conventional PE in FHP. Longer follow-up is required to evaluate whether the oxidation protection with VEPE is associated with lower wear rates and subsequently decreases the rate of THA revisions in the long term.

## Study limitations

The data used in this study was obtained from several studies estimating the FHP between the VEPE and conventional PE liners in THA. The techniques and material were similar but not identical. Another source of limitation was the low number of patients who were included in each cohort. Recommend RCTs with a large volume of patients.

## Conclusion

In terms of FHP, this metanalysis proves better short-term results of the VEPE compared with the conventional PE. A longer follow-up period is required to evaluate whether the oxidation protection gained by Vitamin E results in lower wear rates than conventional PE in the long term.

## Conflict of interest

The authors declare no conflicts of interest.

## Funding

The authors received no specific funding for this work.

## Authors contribution

HE is the main author of this study. He planned the work design, data, analysis/interpretation, and writing the manuscript. MR and HV also contributed to selecting the included studies during the process of screening. They also were involved in study design, statistical analysis. SG and JH played an essential role in this study. They provided the necessary guidance and mentorship.
